# Performance of the Baseline Sport Concussion Assessment Tool in Male and Female Spanish Amateur Rugby Players

**DOI:** 10.3390/biomedicines13020419

**Published:** 2025-02-10

**Authors:** Cristian Solís-Mencía, Juan José Ramos-Álvarez, José Luis Maté-Muñoz, Juan José Montoya-Miñano, Laura Martín, Pablo García-Horcajo, Carlota Requeno-Conde, Elena Oliva-Iglesias, Luis De Sousa-De Sousa, Pablo García-Fernández

**Affiliations:** 1 Department of Medicine, Faculty of Health Sciences, University of Deusto, 48007 Bilbao, Spain; c.solis@deusto.es; 2 Department of Radiology, Rehabilitation and Physiotherapy, School of Sport Medicine, Faculty of Medicine, Complutense University Madrid, 28040 Madrid, Spain; jjmontoy@ucm.es; 3 Department of Radiology, Rehabilitation and Physiotherapy, Faculty of Nursing, Physiotherapy and Podiatry, Complutense University of Madrid, 28040 Madrid, Spain; jmate03@ucm.es (J.L.M.-M.); luisdeso@ucm.es (L.D.S.-D.S.); pablga25@ucm.es (P.G.-F.); 4 Complutense Cisneros Rugby Club, 28040 Madrid, Spain; laury.martin@gmail.com (L.M.); pgarho@gmail.com (P.G.-H.); carlotarequeno1993@gmail.com (C.R.-C.); elenaolivaiglesias7188@gmail.com (E.O.-I.)

**Keywords:** rugby, SCAT, concussion, pre-season, brain injury

## Abstract

**Background/Objectives**: The Sport Concussion Assessment Tool (SCAT) is a test used to screen for suspected concussions, with the results compared to baseline values. If current baseline values are unavailable, they can be compared to baseline values obtained from professional rugby players. The aim of this study was to evaluate the baseline SCAT values in Hispanic community rugby players of both sexes. This cohort study used an observational, prospective, and descriptive design. **Methods**: Participants: A total of 81 female (age: 23.3 ± 3.3 years) and 138 male (age: 23.7 ± 4.3 years) Spanish rugby players who participated in national-level competitions. Interventions (or assessment of risk factors of independent variables): The SCAT was administered as part of the pre-season medical testing, including symptoms endorsed, cognitive submode performance, and balance performance. **Results**: Most of the Spanish community rugby players presented some symptom in the SCAT (male = 75.4%; female = 91.4%). The number and severity of the symptoms reported by the male players were lower than those reported by the female players (*p* = 0.001). The time to complete the tandem gait test and balance test showed differences between sexes (*p* < 0.001). **Conclusions**: The baseline SCAT values of Spanish community rugby players differ from those of professional players, leading to the recommendation of conducting the SCAT for all players before the beginning of the season. If baseline evaluations cannot be performed, the results obtained could serve as a basis for developing reference values for community rugby in the Hispanic population. Recording the menstrual cycle phase during which the SCAT is performed may help improve its interpretation.

## 1. Introduction

Concussion was defined in the 6th International Conference on Concussion in Sport as a “traumatic brain injury caused by a direct blow to the head, neck or body resulting in an impulsive force being transmitted to the brain that occurs in sports and exercise-related activities. This initiates a neurotransmitter and metabolic cascade, with possible axonal injury, blood Flow change and inflammation affecting the brain. Symptoms and signs may present immediately, or evolve over minutes or hours, and commonly resolve within days, but may be prolonged. No abnormality is seen on standard structural neuroimaging studies (computed tomography or magnetic resonance imaging) but in the research setting, abnormalities may be present on functional, blood flow or metabolic imaging studies” [1]. Concussion is frequent in contact sports, with rugby being one of the sports with the highest rates of concussion [2].

The tackle is the game action that generates the most concussions [3], with higher risk for the tackler [4]. This risk increases if the tackler positions their head in front of the player being tackled [5], if the tackler accelerates prior to the tackle [4], and if the tackle is high [6].

Concussion rates in rugby players range between 0.19 and 17.1 per 1000 match hours, with the highest rates occurring in the sub-elite level [7]. Acute concussion has been associated with a greater risk of muscular injury risk, as a consequence of lower motor control after concussion [8]; in exceptional cases, following a new brain injury soon after the concussion, a second impact may take place, which could lead to a fatal outcome [9]. Moreover, in some people, concussion may persist with symptoms for over 2 weeks, with headache being the main symptom, and it has been reported that up to 0.21% of patients may present post-concussion headache for over 3 months [10]. Regarding the long-term effects of concussion, repeated concussions in single person have been associated with chronic traumatic encephalopathy (CTE), although this association has not been fully demonstrated [11,12]. Early diagnosis and the adequate management of concussion allow for a shorter recovery time, reducing the risk of complications or other brain and musculoskeletal injuries [13].

World Rugby recommends that, in cases of suspected concussion, the player should be permanently removed from the game or training and evaluated by a doctor. In this evaluation, the doctor will administer neuropsychological tests. For instance, the Sport Concussion Group designed a measurement instrument for the evaluation of concussions in sports, i.e., the Sport Concussion Assessment Tool (SCAT). The SCAT has two different sections, one that is conducted on the pitch, including the Glasgow scale, and the second that is carried out outside the field, evaluating symptoms, cognitive functions (orientation, immediate-delayed memory, and concentration), and neurological function (including balance). To interpret this test, it is necessary to have previous values, which are not available in community or non-professional rugby [14]. To minimize this problem, World Rugby suggests using normative values obtained from professional players [15]. After comparing the SCAT results with reference values and through clinical judgment, the diagnosis or exclusion of concussion is determined. Having appropriate previous values for a specific population will help reduce the incidence of false negatives. Once concussion is diagnosed, the player must refrain from exercise for 14 days for those under 19 years old and 12 days for adults. Subsequently, there is a gradual increase in the physical and specific demands of rugby before returning to symptom-free training [1,16]. In the evaluation of the concussion, the assessment of the cognitive function is essential, which can be carried out through batteries of neuropsychological tests. The fifth version of the SCAT, or SCAT5, has been used until now [17]; recently, the sixth version (SCAT6) was published [18]. In this new version, the on-field evaluation in the event of an acute injury and the evaluation out of the field have been modified. The off-field evaluation is usually carried out to report baseline values, and it has incorporated an optional evaluation of dynamic balance associated with a cognitive task while it is being carried out. The new version also modified the athlete’s reading of the symptoms, uses only 10 words in immediate-delayed memory, and includes a time component in the concentration assessment [19].

World Rugby, in its player protection policies, has included the use of SCAT adapted for brief completion outside of the pitch in professional rugby. This protocol for the evaluation of brain injuries is called Head Injury Assessment (HIA), and it is a process that includes assessments to be conducted during the match, immediately after the match, and 48 h after the match [15]. To facilitate the HIA evaluation, different studies have been conducted on professional players to determine the baseline SCAT values, which are used as reference values [20]. For community rugby, this evaluation process is not applicable, although SCAT is usually employed to discard or confirm suspected cases of concussion. Due to the time required to complete the SCAT, in many cases, there are no baseline values for community rugby players [14]; consequently, the reference values obtained by World Rugby in professional players are used [15,20]. In community rugby in Spain, there are no reference values to assist us in evaluating the results of the SCAT by comparing them with the baseline data of the players at the beginning of the season; in addition, the SCAT reference results may vary depending on the sport, the level of competition, and the characteristics of the population. Therefore, as World Rugby points out, if there are no reference values, the values of professional players could be used [20]. We consider that it is necessary and of great importance to have specific reference values of the study population for a correct interpretation of the test results. The recommendations of the latest consensus on concussion in sports highlighted, on one hand, the lack of studies in populations outside Anglo-Saxon countries with different cultural contexts, and on the other hand, the lack of information comparing SCAT data between genders [1]. To our knowledge, there are no studies that evaluate baseline SCAT values in the Hispanic community or non-professional rugby players of both sexes, so the reference values of professional players have usually been used in the treatment of concussion in these categories. Therefore, the present study aimed to evaluate and establish baseline SCAT values in Hispanic community rugby players of both sexes, comparing these values between sexes and also exploring the differences in SCAT results between players with and without a history of concussion, thus contributing to a more adequate management of concussions and return to play, making the practice of rugby a safer sport.

## 2. Materials and Methods

### 2.1. Research Design

This cohort study used an observational, longitudinal, prospective, and descriptive design, approved by the Ethics Committee of the University Alfonso X. Madrid, Spain (23 June 2019).

### 2.2. Participants

The sample was constituted by 81 female players and 138 male players who competed at the national level in Spain for three consecutive seasons (2019–2022). The players performed three rugby training sessions per week of 90 min each, plus five gym training sessions of 60 min for men and three sessions of the same duration for women.

### 2.3. Instruments

The paper-format SCAT was administered to all participants during the pre-season, in the general physical preparation phase, at the medical facilities of the club in the afternoon. The data were gathered by the same professional, with the aim of minimizing the bias that could occur in the realization of the tests. Players with a prior history of lower-limb injuries underwent the SCAT assessment only after achieving complete recovery (11:M; 7:F), ensuring that the balance assessment results within the SCAT were not affected. Furthermore, in alignment with the methodology described in previous studies [21,22], none of the players had experienced a concussion within the three months preceding the baseline SCAT evaluation. Each participant also provided demographic information including medical background, rugby position played, cumulative number of years they have played rugby, and previous concussion history.

**Figure 1:**
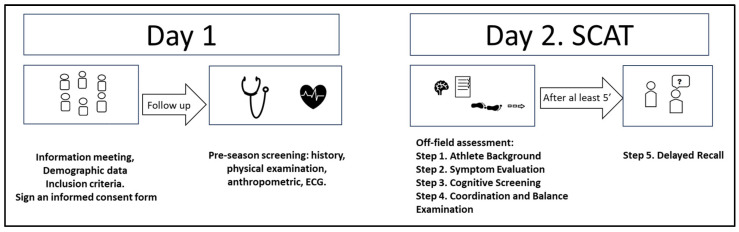
Schematic diagram.

The SCAT is a neurocognitive tool composed of immediate assessment and off-field assessment. Immediate assessment is not required at baseline. The off-field assessment has five steps:

Step 1: Background information on the athlete.
Step 2: Subjective symptom evaluation includes 22 items representing common concussion symptoms, graded on a scale from 0 (none) to 6 (severe), based on how the athlete is currently feeling. The symptom number score is of a possible 22 points, while the symptom severity score is of a possible 132 points.
Step 3: Cognitive screening (based on standardized assessment of concussion; SAC). The SAC is a measure of cognitive functioning that assesses 4 domains: (1) orientation to time, (2) immediate memory using the 10-word list, (3) concentration, and (4) delayed recall. Responses on the SAC are dichotomous (i.e., 0 = incorrect; 1 = correct) and result in a score between 0 and 30, with lower scores representing greater cognitive deficits.
Step 4: Neurological screen includes neck examination, the modified Balance Error Scoring System (m-BESS) Examination, and a Coordination Examination.
Step 5: Delayed recall is part of the SCA and should be performed after 5 min have elapsed since the end of the immediate memory section (Figure 1).

All players voluntarily agreed to participate in the study and signed an informed consent form before participating in it. Each player was informed about the objectives of the study, the steps to be followed, and the methodology to collect the data. The study was conducted in accordance with the Declaration of Helsinki for research involving human beings [23] and in accordance with ethical standards in research in sports and exercise sciences [24]. Data were treated in compliance with Organic Law 3/2018, of 5 December, on the Protection of Personal Data and guarantee of digital rights.

Anthropometric and demographic data of the players were gathered, as well as their medical histories. The instruments used for anthropometric measurements consisted of a Holtain^®^ wall stadiometer (precision ± 1 mm) and a Seca^®^ weight scale (precision ± 0.1 kg). These data, along with those collected in the paper-format SCAT, were recorded in an adapted Excel sheet.

### 2.4. Statistical Analysis

The statistical analysis was performed with the SPSS v.25 software for Windows (IBM SPSS: Statistical Package for Social Science, Chicago, IL, USA). The analysis of the descriptive variables was expressed in means and standard deviations (SD); 95% confidence intervals (CI); median, minimum, and maximum; and 5th and 95th percentiles. The assumption of normality or analysis of the distribution of all continuous variables was verified using the Kolmogorov–Smirnov test (*n* > 50) and the visualization of the histograms, classifying them for subsequent analysis into parametric and non-parametric.

In the comparative analysis, the Mann–Whitney U test was used for the quantitative variables of non-parametric distribution and Student’s t test of independent samples was performed for the quantitative variables of parametric distribution, according to Levene’s test for equality of variances. The statistical differences in the qualitative and categorical variables were calculated using the Chi-Square test (χ^2^) or Fischer’s exact test when more than 20% of the cells presented a value less than five. The effect size for Student’s t test was determined using Cohen’s d, establishing a small effect size for d values between 0.20 and 0.49; medium for values between 0.50 and 0.79; and large for values greater than or equal to 0.80 [25]. The calculation of the effect size of the Mann–Whitney U test was performed with the formula r = Z/√*n* [26]. The effect size for the Chi-Square test (χ^2^) was determined using the PHI Coefficient (φ), which ranges from −1 to +1, where ±1 is perfect association or dissociation, and 0 indicates no association. The significance level was set at *p* < 0.05.

## 3. Results

The mean age of the male and female players was 23.7 and 23.3 years, respectively (Table 1). The rates of previous concussion were 28.4% and 52.18% in the female and male players, respectively. Most of the players stated that they had no health problems, with only 8.69% of the male players and 35.8% of the female players requiring the use of some drug.

Table 2 presents the summary of the SCAT baseline evaluation by sex, showing that the number of symptoms reported by the male players was lower (mean score = 3; min–max: 0–21) than that of the female players (mean score = 5; min–max: 0–18), with statistically significant differences between sexes (*p* = 0.001). In the case of the male players, 24.6% of them reported no symptoms, and in the case of the female players, only 8.6% presented no symptoms. Regarding the severity of the symptoms, statistically significant differences were identified (*p* = 0.001), with the male players presenting lower severity (mean score = 4; min–max: 0–32) than the female players (mean score = 8; min–max: 0–35). In the men, the most frequent symptoms were anxiety or nervousness (43.5%), neck pain (40.6%), and fatigue or low energy (39.1%); the women reported the same symptoms, although in different proportions: fatigue or low energy (56.8%), neck pain (48.1%), and anxiety or nervousness (44.4%) (Table 3).

The time to complete the tandem gait test showed differences between sexes (*p* < 0.001), with the male players requiring less time (10.87 s; 95% CI: 10.58–11.17) than the female players (11.91 s; 95% CI: 11.62–12.19). A total of 98% of the male and female players completed this test in less than 14.4 and 14.0 s, respectively.

The results of immediate memory did not present differences between men and women (*p* = 0.125), with the men obtaining an average value of 22.34 (min–max: 22.0–25.0) and the women obtaining an average value of 23.08 (min–max: 20.0–27.0). Only 2.17% of the male players achieved the maximum score in immediate memory, and none of the female players achieved this maximum score. A total of 98% of the male players obtained a score of 15 or higher out of 30, whereas 98% of the female players obtained a score of 16 or higher out of 30. In delayed memory, no differences were obtained by sex (*p* = 0.563), showing averages of 7.32 and 7.52 (min–max: 6.0–9.0) in men and women, respectively, with 98% of the men being able to remember 2 or more words out of 10, and the women remembering 4 or more words out of 10.

The mean concentration score was similar in the male and female players, who obtained 2.92 and 2.95, respectively (min–max: 2.0–4.0 males; min–max: 2.2–4.0 females), with no differences between sexes (*p* = 0.471). A total of 5.07% and 3.7% of the male and female players, respectively, achieved the maximum score.

The balance errors did not present differences between sexes (*p* = 0.175), with an average of 3.95 in the males and 3.23 in the females. Only 11.5% and 17.28% of the male and female players, respectively, did not present errors in the balance tests, and 2% of the male and female players showed 12.4 and 10.3 errors.

In the comparison between male players with and without a history of concussion, no differences were observed in the number or severity of symptoms, orientation, immediate or delayed memory, concentration, tandem gait, or balance (Table 4). In the case of women with and without a history of concussion, the comparison only revealed differences in balance (*p* = 0.001), with those who had suffered a concussion obtaining an average of 3.85 balance errors (95% CI: 3.1–4.61) compared to the average of 1.69 balance errors (95% CI: 0.74–2.64) in those who had not suffered a concussion (Table 5).

## 4. Discussion

To the best of our knowledge, this is the first study to evaluate the baseline SCAT results in Hispanic community rugby players of both sexes. Therefore, the results obtained could serve as references in Hispanic community rugby. The results of the present study show that most of the participants had some symptoms when the evaluation was conducted, with the female players reporting a larger number of symptoms, with greater severity. The SCAT score, delayed memory, and balance errors were similar in male and female players, although the tandem gait times were longer in the female players. No differences were observed in symptoms, SCAT score, delayed memory, or tandem gait time in players with a history of concussion, who only presented a larger number of balance errors.

The presence of symptoms in most of the male and female players has been previously described in other studies, both in community rugby players [21] and in adolescent rugby players [27]. However, different results have been reported in professional rugby, with 0 and 1 symptoms on average in men and women, respectively [20], which is different from the findings of the present study, where both men and women presented a larger number of symptoms. This difference could be due to the dedication level of the players, with previous studies describing that community rugby players and university athletes present more symptoms and greater severity than professional players [20,21,22,27,28]. Many of our players were undergoing university degrees, where the combination of academic work and the pressure of evaluations may result in higher levels of anxiety, as has been described in previous studies conducted on university students [29]. Previous studies in university athletes as well as in healthy non-professional sub-Saharan athletes have shown a prevalence of muscular pain between 25% and 40%, with the cervical region being one of the areas where a higher prevalence is observed, which is consistent with what we have observed [30,31].

Neck pain was one of the symptoms reported by the participants, which could be due to the fact that the test was carried out during the pre-season phase, i.e., when strength training sessions in the gym are prioritized. This training can lead to neck pain through delayed onset muscle soreness (DOMS) due to neck musculature strengthening work, as it has been observed that the risk of concussion decreases with the improvement in neck musculature strength, and thus neck musculature strength training has been intensified [32]. Another possible reason for these differences in our players is the fact that most professional players are of Anglo-Saxon origin, and the scientific literature has highlighted that there are differences in SCAT results between ethnic groups [21,33]. On the other hand, professional players are more familiar with the use of the Baseline Sport Concussion Assessment Tool than community players, which could influence the results as suggested by the scientific literature. Finally, the differences may be due to the way this assessment is performed in the SCAT5, where athletes must read the instructions and the symptom list while assigning scores to each item, which in the SCAT6 has been modified [18,19], and it will be interesting to see how these values can be modified in this population.

In the analysis by sex, our results are in line with those of other studies, which show that female players present a larger number of symptoms and greater severity [20,21,27,34,35]. After a concussion, it has been observed that male players report fewer symptoms than female players to avoid exclusion from training sessions or matches [36]; in our study, we prevented such bias, since all determinations were performed during the pre-season phase. The greater number of symptoms and greater severity in the case of female players may be related to the phase of the menstrual cycle in which they are at the time of the evaluation. In a recent study in female rugby players, more than 90% presented symptoms such as anxiety/irritability or fatigue and low energy related to their menstrual cycle [37]. However, in our study, the stage of the players’ cycle at the time of their evaluation was not recorded as it was not required in the SCAT. In a recent review, it has been described that between 74 and 83% of athletes present some symptoms such as anxiety/irritability, fatigue/low energy, or headache during their menstrual cycle [38], raising the need to incorporate the moment of the menstrual cycle in which the evaluation is performed in the SCAT. The fact that it was found that players without injury at the time of the test reported symptoms highlights a need for the clinical judgment of the physician to determine the cause of these symptoms and should be kept mind when determining the recovery of a player who has suffered a concussion.

Regarding the SCAT scores and the balance errors, there were no differences between sexes, which is in disagreement with the findings of other studies [20,21,22,28,33]. Differences were only observed in the tandem gait time, which was longer in the female players, in line with the results of studies conducted in community and professional rugby players [20,21]. The reason for this difference is not known, but it could be due to the size of the foot; having a larger foot size in men would imply a lower number of steps and therefore a shorter time to complete the walk.

Regarding immediate memory, our participants obtained higher scores than community and professional rugby players, which could be due to the academic training of the former, most of whom were studying at university or had completed higher education [20,21]. Concerning delayed memory, the average values of the women were higher than those reported in previous studies with community rugby players [21] and university athletes [33], whereas the average values of the men were also higher than those of community rugby players and university athletes [21,33] and slightly higher than those of professional rugby players [20]. On one hand, the average orientation values were similar, in both sexes, to those observed in community and professional rugby [20,21]. On the other hand, the average concentration values of our participants were lower than those of community and professional rugby players [20,21] and university athletes [28]. Lastly, the average values of balance errors were higher than those observed in professional players for both men and women [20], and lower than those of female university athletes [20,28].

Regarding the players with a history of concussion, differences were only found in women in terms of balance, which is not in line with previous studies in community rugby players, which report no alterations in this parameter. However, unlike in our study, lower results have been presented in immediate memory and concentration in professional players [21] and university athletes [28] with a history of concussion.

It has been described that, after a concussion, recovery from the symptoms occurs before neurocognitive recovery [39]. A study conducted on rugby players between 14 and 18 years of age reported an alteration in dynamic balance in players with a history of concussion [40], although no alteration was observed in static balance, which is in line with the findings of our study. Previous studies have demonstrated an increase in the risk of sustaining musculoskeletal injuries in athletes after a concussion [41,42], possibly due to deficient motor control after the concussion [36], which can be objectified by the balance alteration observed in athletes who have suffered a concussion. However, further research is required to describe the duration of these alterations and other influential factors (e.g., sex), since it has been highlighted that recovery is longer in women. It would be interesting to carry out longitudinal studies that compare the increase in musculoskeletal injuries in players of both sexes who have suffered a concussion and present balance alterations in the SCAT.

The main strength of this study is that it is the first study carried out in Spain in community rugby players, establishing reference values using the Sport Concussion Assessment Tool, enabling the diagnosis and prevention of complications associated with concussion associated with sport, making the return to play of players with possible concussion safer.

The primary limitation of this study arises from the relatively small number of players included. This athlete population, particularly within the context of Hispanic community rugby, has not been extensively studied, as emphasized in the latest medical conferences on the subject [1]. As previously discussed, the unique characteristics of this population, combined with the practical challenges of administering concussion assessments, make it difficult to apply the test to larger community rugby groups. This complexity highlights the need for ongoing research into the development of new tools that can assess concussions in a more streamlined, simple, and objective manner, ensuring that these tools can be used effectively across the full spectrum of rugby players, regardless of their skill level or competition tier. Another significant limitation of this study relates to the absence of data on the menstrual cycle of female players, which prevented us from exploring its potential influence on concussion outcomes. The menstrual cycle is known to affect various physiological factors that could impact concussion recovery and symptom presentation, and thus failing to account for it introduces a gap in the results. Moving forward, it would be critical to include the phase of the menstrual cycle in future concussion assessment tools, especially for rugby, in order to fully understand and study its influence on concussion results and recovery times. Addressing this factor could significantly enhance the precision and relevance of concussion assessments for female athletes.

## 5. Conclusions

It was observed that most of the Spanish community rugby players who participated in this study presented some symptom when they performed the baseline SCAT, with the women reporting more symptoms and greater severity. Moreover, they required more time than men to complete the tandem gait test. In relation to immediate memory, delayed memory, orientation, concentration, and balance errors, there were no differences between sexes in the baseline SCAT. The history of concussion only affected static balance in women. The SCAT baseline values of Spanish community rugby players are different from the baseline SCAT values of professional players, which leads to the recommendation of conducting the SCAT in all players before the beginning of the season, regardless of the game level. The results obtained could serve as a basis for developing reference values for community rugby in the Hispanic population. Additionally, recording the menstrual cycle phase during which the SCAT is performed may improve its interpretation, as symptoms could be linked to this phase.

## Figures and Tables

**Table 1:** Descriptive data measurements for anthropometric variables, age, and previous concussions.

	Men (*n* = 138)	Women (*n* = 81)
	Mean ± SD	Mean ± SD
Age	23.7 ± 4.3	23.3 ± 3.3
Weight (kg)	89.8 ± 13.7	67.0 ± 10.2
Height (cm)	180.3 ± 6.1	166.5 ± 6.4
Years playing rugby	11.6 ± 4.1	5.8 ± 2.7
Previous concussions	*n* (%)	*n* (%)
0	66 (47.82)	58 (71.60)
1	36 (26.00)	9 (11.11)
2	19 (13.76)	10 (12.34)
3	6 (4.34)	2 (2.46)
> 3	10 (7.24)	2 (2.46)
Hospitalized for prior head injury?		
No	109 (79.0)	70 (86.4)
Yes	29 (21.0)	11 (13.6)
Diagnosed or treated for headache disorder or migraines?		
No	129 (93.5)	66 (81.5)
Yes	9 (6.5)	15 (18.5)
Diagnosed with a learning disability or dyslexia?		
No	131 (94.9)	75 (92.6)
Yes	7 (5.1)	6 (7.4)
Diagnosed with attention-deficit hyperactivity disorder?		
No	134 (97.1)	75 (92.6)
Yes	4 (2.9)	6 (7.4)
Diagnosed with depression, anxiety, or other psychiatric disorder?		
No	135 (82.6)	68 (90.1)
Yes	3 (17.4)	13 (9.9)
Pharmacotherapy		
No	126 (75.4)	52 (93.8)
Yes	12 (24.6)	29 (6.2)
Symptoms worsen with physical exercise?		
No	114 (97.8)	73 (84.0)
Yes	24 (2.2)	8 (16.0)
Symptoms worsen with intellectual activity?		
No	104 (91.3)	76 (64.2)
Yes	34(8.7)	24(35.8)

**Table 2:** Summary of symptom score data stratified by sex.

Summary Statistics	Men (*n* =138)	Women (*n* = 81)	*p* Value	Effect Size
Total No. of Symptoms (of 22)				
Median (min–max)	3 (0–21)	5 (0–18)	0.001 ^†^	0.39
Mean ± SD; 95% CI	4.32 ± 4.47; 95% CI 3.52–5.11	6.02 ± 4.50; 95% CI 5.02–7.02		
Percentile (25–75)(10–90)(2–98)	(0.7–7.0)(0.0–10.0)(0.0–16.6)	(2.0–9.0) (1.0–12.8) (0.0–16.0)		
Total severity score (of 132)				
Median (min–max)	4 (0–32)	8 (0–35)	0.001 ^†^	0.37
Mean ± SD; 95% CI	6.94 ± 7.95; 95% CI 5.53–8.35	10.03 ± 8.62; 95% CI 8.11–11.95		
Percentile (25–75)(10–90)(2–98)	(0.7–10.0)(0.0–20.2)(0.0–31.0)	(3.0–14.0)(1.0–22.0)(0.0–33.0)		
Tandem test completion time				
Median (min–max)	11 (7.6–16.0)	11.93 (9.42–18.0)	<0.001 *	0.66
Mean ± SD; 95% CI	10.87 ± 1.67; 95% CI 10.58–11.17	11.91 ± 1.26; 95% CI 11.62–12.19		
Percentile (25–75)(10–90)(2–98)	(9.5–12.0)(9.0–13.4)(7.6–14.4)	(10.9–12.9)(10.2–13.6)(9.4–14.0)		
Orientation				
Median (min–max)	5 (0–5)	5 (3–5)	0.352 ^†^	0.16
Mean ± SD; 95% CI	4.75 ± 0.93; 95% CI 4.58–4.91	4.62 ± 0.34; 95% CI 4.84–5.0		
Percentile (25–75)(10–90)(2–98)	(5.0–5.0)(4.5–5.0)(0.0–5.0)	(5.0–5.0)(5.0–5.0)(3.0–5.0)		
Lazy Memory 10				
Median (min–max)	8 (0–10)	8 (4–10)	0.563 ^†^	0.13
Mean ± SD; 95% CI	7.32 ± 2.07; 95% CI 6.96–7.68	7.51 ± 1.75; 95% CI 7.12–7.90		
Percentile (25–75)(10–90)(2–98)	(6.0–9.0)(4.0–10.0)(2.0–10.0)	(6.0–9.0)(5.0–10.0)(4.0–10.0)		
Deferred Memory 30				
Median (min–max)	22 (15–30)	24 (16–29)	0.125 ^†^	0.22
Mean ± SD; 95% CI	22.34 ± 3.45; 95% CI 21.73–22.95	23.08 ± 3.79; 95% CI 22.24–23.93		
Percentile (25–75)(10–90)(2–98)	(20.0–25.0)(17.1–27.0)(15.0–30.0)	(20.0–27.0)(18.0–28.0)(16.0–29.0)		
Concentration				
Median (min–max)	3 (0–5)	3 (1–5)	0.471 ^†^	0.06
Mean ± SD; 95% CI	2.92 ± 1.42; 95% CI 2.66–3.17	2.95 ± 0.99; 95% CI 2.72–3.17		
Percentile (25–75)(10–90)(2–98)	(2.0–4.0)(0.0–4.0)(0.0–5.0)	(2.2–4.0)(1.0–4.0)(1.0–5.0)		
Balance Errors				
Median (min–max)	3 (0–15)	2 (0–11)	0.175 ^†^	0.07
Mean ± SD; 95% CI	3.95 ± 3.16; 95% CI 3.39–4.51	3.23 ± 2.82; 95% CI 2.60–3.86		
Percentile (25–75)(10–90)(2–98)	(1.0–6.0)(0.0–8.1)(0.0–12.4)	(1.0–6.0)(0.0–7.0)(0.0–10.3)		

* Student’s t test was used. † Mann–Whitney U test was used.

**Table 3:** Proportion and association analysis of the initial SCAT of men and women reporting each symptom.

Symptoms	Men (*n* = 138)	Women (*n* = 81)	*p* Value	Efect Size
Headache	28.8%	38.3%	0.010 *	−0.21
Head pressure	13.8%	23.5%	0.158 *	−0.12
Neck pain	40.6%	48.1%	0.355 *	−0.07
Nausea or Vomiting	0.7%	7.4%	0.045 ^†^	−0.18
Dizziness	6.5%	9.9%	0.265 ^†^	−0.06
Blurry Vision	8.0%	9.9%	0.726 *	−0.03
Balance problems	7.2%	17.3%	0.022 *	−0.15
Hypersensitivity to Light	11.6%	24.7%	0.031 *	−0.17
Noise hypersensitivity	4.3%	13.6%	0.032 ^†^	−0.16
Feeling Slow	13.0%	11.1%	0.417	0.02
Feeling Dazed	5.1%	13.6%	0.027 ^†^	−0.15
I don’t feel well	18.8%	24.7%	0.233 *	−0.06
Difficult to focus	32.6%	55.7%	0.054 *	−0.13
Difficulty remembering	23.2%	35.8%	0.044 *	−0.13
Fatigue or Lack of Energy	39.1%	56.8%	0.033 *	−0.17
Confusion	8.7%	4.9%	0.141 *	0.07
Drowsiness	34.8%	30.9%	0.474 *	0.04
Difficulty due to Fatigue	26.1%	42.0%	0.031 *	−0.16
More Emotional	18.8%	33.3%	0.004 *	−0.16
Irritability	25.4%	30.9%	0.236 *	−0.06
Sad	22.3%	28.4%	0.003 *	−0.20
Nervous or Anxious	43.5%	44.4%	0.169 *	−0.00

* The Chi-Square test (χ^2^) was used. † Fisher’s exact test was used.

**Table 4:** Summary of symptom score data for men.

Summary Statistics	Men Not Concussed (*n* = 72)	Men Concussed (*n* = 66)	*p* Value	Efect Size
Total No. of Symptoms (of 22)				
Median (min–max)	4 (0–13)	3 (0–21)	0.239 ^†^	0.20
Mean ± SD; 95% CI	3.81 ± 3.32; 95% CI 2.99–4.63	4.80 ± 5.41; 95% CI 3.4–6.19		
Percentile (25–75)(10–90)(2–98)	(0.25–6.0)(0.0–9.0)(0.0–13.5)	(0.75–8.0)(0.0–14.0)(0.0–20.3)		
Total severity score (of 132)				
Median (min–max)	4 (0–24)	4 (0–32)	0.316 ^†^	0.17
Mean ± SD; 95% CI	6.0 ±6.16; 95% CI 4.48–7.54	7.83 ± 9.43; 95% CI 5.41–10.25		
Percentile (25–75)(10–90)(2–98)	(0.25–10.0)(0.0–18.4)(0.0–27.7)	(0.75–11.25)(0.0–24.3)(0.0–31.6)		
Tandem test completion time				
Median (min–max)	11.0 (7.6–16)	10.7 (10.3–11.1)	0.266 *	0.19
Mean ± SD; 95% CI	11.0 ± 1.76; 95% CI 10.6–11.4	10.7 ± 1.60; 95% CI 10.3–11.1		
Percentile (25–75)(10–90)(2–98)	(9.7–12.1)(9.0–13.3)(7.6–15.7)	(9.3–12.0)(8.9–13.5)(7.8–14.0)		
Orientation				
Median (min–max)	5 (0–5)	5 (0–5)	0.456 ^†^	0.07
Mean ± SD; 95% CI	4.78 ± 0.89; 95% CI 4.56–5.00	4.65 ± 1.07; 95% CI 4.37–4.93		
Percentile (25–75)(10–90)(2–98)	(5.0–5.0)(5.0–5.0)(0.0–5.0)	(5.0–5.0)(4.0–5.0)(0.0–5.0)		
Lazy Memory 10				
Median (min–max)	8 (0–10)	8 (2–10)	0.527 ^†^	0.11
Mean ± SD; 95% CI	7.24 ± 2.18; 95% CI 6.70–7.78	7.39 ± 1.87; 95% CI 6.91–7.87		
Percentile (25–75)(10–90)(2–98)	(6.0–9.0)(4.0–10.0)(0.7–10.0)	(6.0–9.0)(4.0–10.0)(2.5–10.0)		
Deferred Memory 30				
Median (min–max)	22 (15–29)	21 (16–30)	0.901 ^†^	0.02
Mean ± SD; 95% CI	22.38 ± 3.60; 95% CI 21.49–23.27	22.21 ± 3.35; 95% CI 21.3–23.07		
Percentile (25–75)(10–90)(2–98)	(20.0–25.0)(17.0–27.2)(15.0–28.6)	(20.0–25.0)(17.4–27.0)(16.0–30.0)		
Concentration				
Median (min–max)	3 (0–5)	4 (0–5)	0.520 ^†^	0.11
Mean ± SD; 95% CI	2.81 ± 1.35; 95% CI 2.47–3.15	3.01 ± 1.49; 95% CI 2.63–3.40		
Percentile (25–75)(10–90)(2–98)	(2.0–4.0)(0.0–4.0)(0.0–5.0)	(2.0–4.0)(0.0–4.0)(0.0–5.0)		
Balance Errors				
Median (min–max)	4 (0–15)	3 (0–12)	0.866 ^†^	0.03
Mean ± SD; 95% CI	3.98 ± 3.42; 95% CI 3.13–4.83	4.04 ± 3.04; 95% CI 3.26–4.82		
Percentile (25–75)(10–90)(2–98)	(1.0–5.7)(0.0–8.7)(0.0–14.5)	(1.7–6.0)(0.7–8.3)(0.0–12.0)		

* Student’s t test was used. † Mann–Whitney U test was used.

**Table 5:** Summary of symptom score data for women.

Summary Statistics	Females Not Cocussed (*n* = 58)	Females Concussed (*n* = 23)	*p* Value	Efect Size
Total No. of Symptoms (of 22)				
Median (range)	4 (0–15)	6 (0–18)	0.064 ^†^	0.37
Mean ± SD 95% CI	4.78 ± 4.02; 95% CI 3.04–6.52	6.52 ± 4.62; 95% CI 5.30–7.75		
Percentile (25–75)(10–90)(2–98)	(2.0–8.0)(1.0–11.0)(0.0-/)	(2.0–10.0)(0.0–14.0)(0.0–17.4)		
Total severity score (of 132)				
Median (range)	5 (0–28)	10 (0–35)	0.093 ^†^	0.48
Mean ± SD 95% CI	7.04 ± 6.73; 95% CI 4.12–9.95	11.24 ± 9.04; 95% CI 8.84–13.64		
Percentile (25–75)(10–90)(2–98)	(3.0–10.0)(1.0–17.6)(0.0-/)	(3.7–18.2)(0.0–23.1)(0.0–34.4)		
Tandem test completion time				
Median (range)	11.8 (9.8–14.0)	12 (9.4–14)	0.643 *	0.11
Mean ± SD 95% CI	11.8 ± 1.3; 95% CI 11.2–12.3	11.95 ± 1.24; 95% CI 11.6–12.2		
Percentile (25–75)(10–90)(2–98)	(10.3–13.0)(10.0–13.9)(0.0-/)	(11.1–12.9)(10.2–13.6)(9.4–14.0)		
Orientation				
Median (range)	5 (3–5)	5 (4–5)	0.506 ^†^	0.34
Mean ± SD 95% CI	4.82± 0.57; 95% CI 4.57–5.07	4.96 ± 0.18; 95% CI 4.91–5.01		
Percentile (25–75)(10–90)(2–98)	(5.0–5.0)(3.8–5.0)(3.0-/)	(5.0–5.0)(5.0–5.0)(4.0–5.0)		
Lazy Memory 10				
Median (range)	8 (5–10)	7 (4–10)	0.425 ^†^	0.21
Mean ± SD 95% CI	7.78 ± 1.53; 95% CI 7.11–8.44	7.40 ± 1.83; 95% CI 6.91–7.88		
Percentile (25–75)(10–90)(2–98)	(7.0–9.0)(5.0–9.0)(5.0-/)	(6.0–9.0)(5.0–10.0)(4.0–10.0)		
Deferred Memory 30				
Median (range)	25 (16–29)	23 (16–29)	0.062 ^†^	0.43
Mean ± SD 95% CI	24.21 ± 4.04; 95% CI 22.46–25.96	22.63 ± 3.62; 95% CI 21.67–23.59		
Percentile (25–75)(10–90)(2–98)	(21.0–28.0)(18.0–28.0)(16.0-/)	(20.0–25.2)(17.0–27.1)(16.1–29.0)		
Concentration				
Median (range)	3 (1–4)	3 (1–5)	0.317 ^†^	0.30
Mean ± SD 95% CI	2.73 ± 1.05; 95% CI 2.28–3.19	3.03 ± 0.96; 95% CI 2.77–3.29		
Percentile (25–75)(10–90)(2–98)	(2.0–4.0)(1.0–4.0)(1.0-/)	(3.0–4.0)(1.8–4.0)(1.0–5.0)		
Balance Errors				
Median (range)	1 (0–9)	4 (0–11)	0.001 ^†^	0.82
Mean ± SD 95% CI	1.69 ± 2.20; 95% CI 0.74–2.64	3.85 ± 2.83; 95% CI 3.10–4.61		
Percentile (25–75)(10–90)(2–98)	(0.0–2.0)(0.0–5.2)(0.0-/)	(1.0–6.0)(0.0–8.0)(0.0–10.8)		

* Student’s t test was used. † Mann–Whitney U test was used.

## Data Availability

The original contributions presented in this study are included in the article. Further inquiries can be directed to the corresponding author.
